# Revolutions at the frontline of multiple myeloma treatment: lessons and challenges to finding a cure

**DOI:** 10.3389/fonc.2025.1578529

**Published:** 2025-06-20

**Authors:** Jeries Kort, Andrea Rivera, Sindhuja Senigarapu, James J. Driscoll

**Affiliations:** ^1^ Division of Hematology and Oncology, Department of Medicine, University Hospitals Cleveland Medical Center, Cleveland, OH, United States; ^2^ Case Comprehensive Cancer Center, Case Western Reserve University, Cleveland, OH, United States; ^3^ Department of Medicine, Ponce Health Sciences University, Ponce, Puerto Rico; ^4^ Heart, Lung Blood Summer Program, Case Western Reserve University, Cleveland, OH, United States; ^5^ Department of Anesthesiology, University Hospitals Cleveland Medical Center, Cleveland, OH, United States; ^6^ Adult Hematologic Malignancies and Stem Cell Transplant Section, Seidman Cancer Center, University Hospitals Cleveland Medical Center, Cleveland, OH, United States

**Keywords:** myeloma, proteasome, immunotherapy, chemoresistance, CAR T cell, plasma cell

## Abstract

Multiple myeloma (MM) is a cancer of bone marrow plasma cells. A noteworthy ensemble of therapies has been introduced over the past quarter century that exert antimyeloma activities through diverse mechanisms and achieve durable disease control in many patients. The discovery that proteasome inhibitors (PIs) and immunomodulatory drugs (IMiDs) target specific plasma cell features that reflect disease biology and exert antimyeloma activity led to transformative changes in treatment algorithms. Recently, advances in immunotherapy have emerged and represent a promising option with the potential to capture immunologic memory and yield more durable responses in MM patients. Idecabtagene vicleucel and ciltacabtagene autoleucel are chimeric antigen receptor (CAR) T-cell immunotherapies that attach to the extracellular domain of the B-cell maturation antigen (BCMA) and have demonstrated significant response rates in heavily-treated patients. These agents are FDA-approved for relapsed and/or refractory (RR)MM patients previously treated with PIs, IMiDs, and CD38-directed monoclonal antibodies. Most patients who receive CAR T-cell therapy relapse after prolonged or brief remission, and a more thorough understanding of the resistance mechanisms following CAR T-cell infusion is needed. Bispecific antibodies (BsAbs) are engineered to simultaneously bind to both cancer and immune cells and trigger a direct tumor-specific cytotoxic response. BsAbs and CAR T-cells are major histocompatibility complex (MHC)-independent approaches to treat MM and do not require T-cell receptor (TCR) specificity. Agents that target BCMA and G protein-coupled receptor class C group 5 member D (GPRC5D) demonstrate impressive clinical responses, while early-phase trials targeting FcRH5 are promising. Here, we provide a comprehensive overview of their individual efficacy, adverse effects, and limitations that impact broader application.

## 1 Introduction

Multiple myeloma (MM) is a plasma cell (PC) malignancy that lacks a cure but can be effectively managed for many years in most patients (1–3). Patients diagnosed with MM in time relapse and become recalcitrant to current treatments (4, 5). Despite significant advances in treatment modalities, myeloma relapse is common and carries a poor prognosis (3–5). The incidence rate is 7/100,000 in the U.S with a median age of onset at 69 years (6). Monoclonal gammopathy of undetermined significance (MGUS) and smoldering MM (SMM) are premalignant PC proliferative disorders that are thought to universally precede the development of MM. MGUS affects ∼3% of individuals >50 years old, and MGUS patients usually do not advance to MM (7). SMM demonstrates high variability, with some cases closely resembling MGUS while others exhibit similarity and comparable risk of MM. Nearly 60% of SMM patients exhibit MM two years post-diagnosis depending on clinical and genetic factors (8). MM as well as the precursor disorders are relatively more common in those of African descent (9–11).

The majority of individuals diagnosed with MM are 65 to 75 years of age. MM is responsible for ~15% of hematological malignancies and ~22% of hematological malignancy-related deaths in the US. Globally, there is a rising trend in the incidence of MM, particularly in males and those >50 years of age, as well as individuals from high-income populations (12). An overall declining global trend of myeloma mortality was more evident in females. Lifestyle, diagnosis capacity, and treatment availability may be modified and improved to control the increasing trends that are observed populations at greater risk (10, 11).

Myeloma cells are predominantly detected in patient bone marrow (BM) but are additionally revealed in the circulatory system and at extramedullary sites, particularly at advanced stages of illness. Myeloma cells generally synthesize, fold, assemble and secrete monoclonal immunoglobulin (Ig) proteins (monoclonal protein, M protein). The IgG isotype accounts for ~50% for MM cases, IgA 20%, while the IgD and IgM phenotypes are observed less frequently (2% and 0.5%). Approximately ~20% of those diagnosed with myeloma secrete only monoclonal κ or λ free light chains (FLC) (light-chain myeloma or Bence Jones myeloma), while some have non- or hyposecretory disease (13–15).

**Figure 1:**
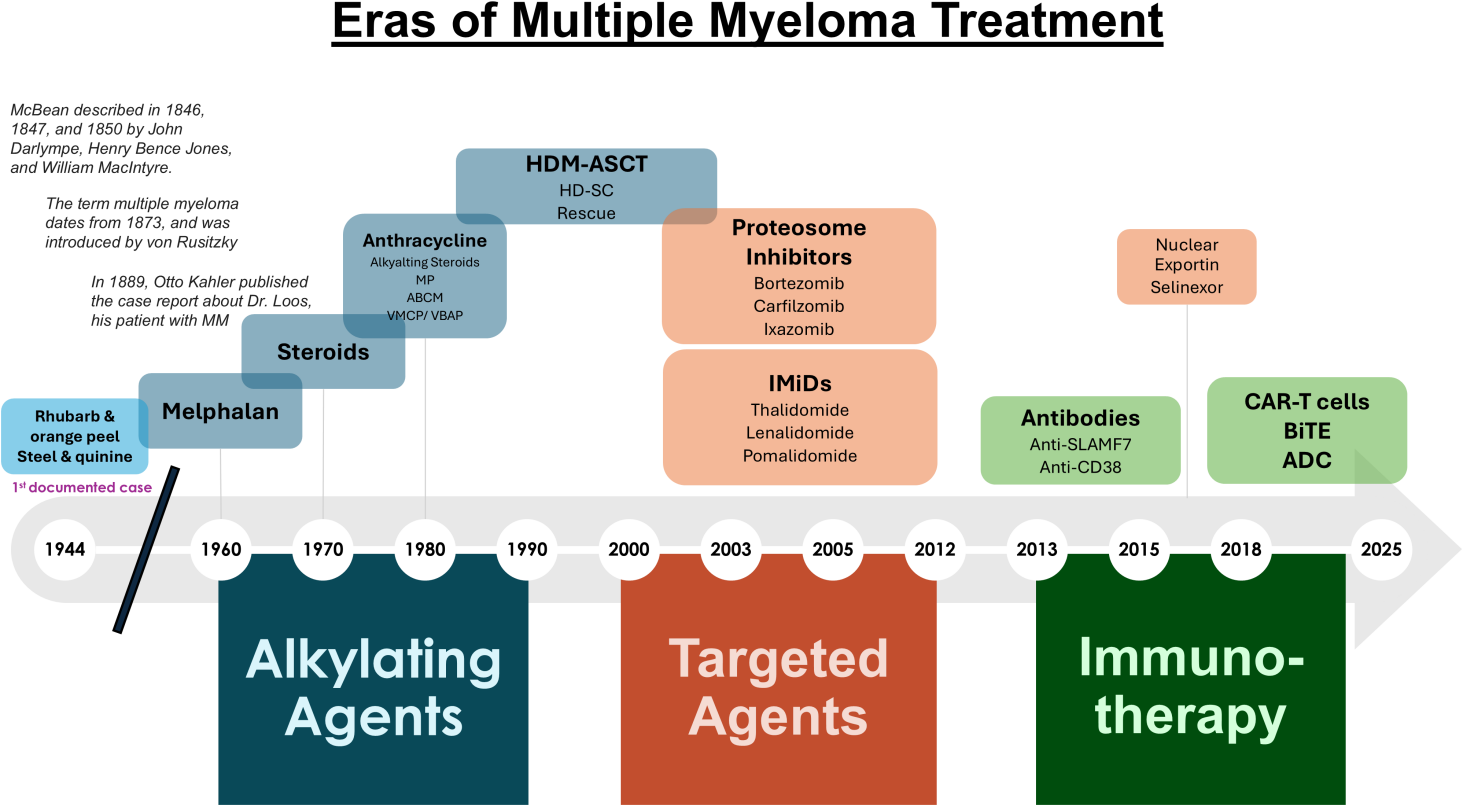
Eras of multiple myeloma treatment. Shown is a timeline of the agents used throughout the past two centuries to treat MM.

Transformative advances in the treatment of MM over the past quarter century has led to a dramatic improvement in patient quality-of-life as well as progression free survival (PFS) and overall survival (OS) (**Figure 1**). A more precise mechanistic appreciation of myelomagenesis and disease features and the cellular, protein and genomic level has led to advances in diagnosis, prognosis, and response assessment, and tremendously informed the discovery of actionable targets and novel agents. Median OS for patients varies with age, eligibility for autologous stem cell transplant (ASCT), tumor cytogenetics, treatment depth and duration of response, and other factors (16). Data from randomized controlled trials using modern therapy show that the median survival in multiple myeloma is approximately 6 years (17). In the subset of patients eligible for ASCT, 4-year survival rates are more than 80% (18); the median OS among these patients is more than 8 years (19, 20). However, not all MM patients have benefited from the vast array of treatment options due to lack of timely access to treatment, inherently therapy refractory disease, and ineligibility to novel therapies. Hence, an improved understanding of timely and effective sequencing of currently approved MM drugs may maximize therapeutic efficacy.

## 2 Earliest recognition on MM

The first widely recognized case of MM was reported in 1844 by Samuel Solly for a highly respectable tradesman from London named Mr. Thomas Alexander McBean (21, 22) Dr. William Macintyre documented attributes that resembled Bence Jones proteinuria. The chemical pathologist Henry Bence Jones confirmed the results of Macintyre (23). The disease was infrequently documented until 1889 with the case report by Kahler (24). The subject of Kahler survived for 8 years despite less than perfect chemotherapy. In 1922, Bayne-Jones and Wilson (25) identified two discrete groups of Bence Jones protein, but a connection between Bence Jones and myeloma serum proteins was not demonstrated until 1956 by Korngold and Lipari (26). The discovery that light chains from a serum IgG myeloma protein and the Bence Jones protein from the same patient’s urine were identical marked a significant breakthrough. Sarah Newbury was a 39-year-old that had developed fatigue, bone pain and fractures (21). At autopsy of the patient, her BM had been replaced by a red substance whose cells were remarkably similar to those found at the autopsy of Mr. McBean.

## 3 Early treatment of MM

Frontline therapy for newly diagnosed MM patients has improved markedly from the rhubarb pill and infusion of orange peel that was given in the 1840’s followed by phlebotomy and application of leeches later administered as “maintenance therapy” (**Figure 1**). In 1947, Alwall described that urethane reduced serum globulin levels, increased hemoglobin, eliminated proteinuria, and decreased BM PCs in a myeloma patient (27). The treatment approach remained the standard for nearly 15 years. Blokhin et al. in 1958 reported the benefit of sarcolysin (melphalan) in 3 of 6 patients with MM (28). Bergsagel et al. in 1962 reported significant improvement in 8 of 24 patients treated with melphalan (29). Holland et al. in 1966 administered urethane or placebo consisting of cherry and cola–flavored syrup to previously treated or untreated patients with promising results (30). Hoogstraten et al. found that melphalan followed yielded responses in 78% of patients that were either newly diagnosed (ND) or had previously treated disease (31).

A placebo-controlled double-blind trial demonstrated that prednisone as a single agent produced significant decreases in serum globulin and an increase in hematocrit but no difference in survival compared with a placebo (32). Two Cancer and Leukemia Group B (CALGB) protocols showed that single agent prednisone produced a 44% objective response rate (ORR) (33). Melphalan combined with prednisone (MelPred) was then established in a randomized trial of MM patients led by Alexanian et al, in which survival was 6 months longer with MP relative to Mel alone (34). In 1974, Lee et al. treated 36 MM patients with carmustine, cyclophosphamide, Mel, vincristine, and Pred (M-2 protocol) and reported that 60% had excellent subjective and objective responses (35). Case et al. later reported an 87% response rate in patients with MM given the M-2 protocol (36). High-dose chemotherapy with Mel 200 mg/m (2) (Mel200) became the conditioning regimen of choice with ASCT and consolidation after induction therapy for suitable patients, prolongs PFS and is recommended upfront for all eligible patients (18, 37).

## 4 Targeting the proteasome revolutionized multiple myeloma treatment

PCs are terminally differentiated B lymphocytes that dwell within the BM. A genotypically and phenotypically discrete population of long-lived PCs (LLPC) has the ability to live for extraordinarily extended periods in humans. Knowledge of LLPC biology impacts myelomagenesis and provides insights into disease treatment. Myeloma shares many tumor intrinsic survival programs as LLPCs. Most PCs are short-lived to limit antibody responses (38). Hence, MM cells are exquisitely sensitive to events that disrupt proteostasis (39–41). PCs differentiate from B lymphocytes to sustain antibody production, and as professional Ab secretors, PCs serve as a model system to dissect proteostasis and the stresses that high volume protein synthesis, folding and transport entail.

As early as the1940’s, it was recognized that all the constituents of an organism are in a constant state of chemical renewal (42). Schoenheimer developed the term “dynamic state of body constituents” to describe the state in which all constituents of the cell are continually degraded and replaced upon new biosynthesis. These thoughts led to the emergence of subsequent seminal discoveries that revealed the destruction of intracellular and cytosolic proteins within the cell is regulated with exquisite selectivity. Ciechanover, Hershko and Rose discovered, studied and revealed the discrete intricacies the ubiquitin (Ub)-mediated proteolytic pathway, a process where an enzymatic system tags unwanted protein substrates with the 76-amino acid polypeptide Ub (43). Ub-tagged proteins are then transported to the proteasome, a large multisubunit proteolytic complex, within which proteins are hydrolyzed to peptides and Ub is recycled. Myriad cellular processes, e.g., protein homeostasis and immunity, are regulated through the Ub pathway. A causal role for defects in the Ub-dependent proteolytic pathway have detected in a number of human diseases, e.g., cancer.

Proteasomes requisite components of the Ub-dependent protein degradation pathway and function as the catalytic core of the pathway to hydrolyze ubiquitinated protein substrates (44, 45). Proteasomes are extremely sophisticated complexes designed to perform the selective, efficient and processive hydrolysis of denatured, misfolded and redundant proteins (46). These structures are dynamic, tightly governed protein degrading, multicomponent complexes that are structurally and functionally conserved throughout evolution. Proteasomes consist of >30 distinct protein subunits and exhibit a MW of ~2.5 MDa. The barrel-shaped 20S proteasome core particle (CP) is formed by axial stacking of 4 heptameric rings: 2 identical inner β-rings, each formed by 7 distinct β-subunits (β_1–7_), and 2 identical outer α-rings each formed by 7 different α-subunits (α_1–7_). A 19S regulatory particle (RP) that recognizes proteins bearing a multi-Ub chain caps either or both end of the 20S CP (46, 47). Proteasomes collaborate with the Ub-conjugating enzymes to play a prominent role in the control of cellular activities by rapidly and unidirectionally catalyzing protein degradation. Constant, highly elevated rates of protein synthesis coupled with the need for highly efficient protein clearance mechanisms make MM cells exquisitely sensitive to the slightest perturbations in proteostasis. Hence, MM offers an opportunity to target this intrinsic survival vulnerability with the administration of PIs. Interestingly, endogenous PIs were also detected in mammalian cell lysates and shown to regulate proteasomal catalytic activities (48, 49). Carfilzomib and ixazomib were later developed as second-generation PIs and have increased patient response rates, survival and safety (50, 51). PIs are a cornerstone of current treatment approaches in MM. While survival outcomes have improved significantly over the past two decades for NDMM patients, elderly patients have not yielded the same magnitude of benefit as evidenced by higher rates of reported myeloma-related deaths in patients >75 years of age (52–54).

## 5 IMiDs

The development of the IMiDs which include thalidomide (Thal), lenalidomide (Len), and pomalidomide (Pom), has contributed significantly to these improved outcomes (55, 56). Len is widely used in the treatment of ND transplant-eligible and transplant-ineligible MM patients, in the maintenance setting post-transplant and in the relapsed/refractory setting, while Pom is currently utilized in the relapsed/refractory setting. IMiDs are a critical component of therapeutic combinations for all stages of the disease and are also used as a single-agent maintenance therapy after ASCT. At diagnosis, most patients are sensitive to IMiD-based combination therapy; however, ~5% are refractory and form an important group with difficult-to-manage disease (56). Patients who are initially sensitive eventually acquire resistance, and IMiD refractory states are associated with shorter PFS and OS in response to subsequent therapies. IMiDs demonstrate a multitude of activities, including anti-angiogenic, cytotoxic, and immunomodulatory. However, the more recent discoveries that the IMiDs bind to cereblon and thus regulate the ubiquitination of key transcription factors including IKZF1 and IKZF3, have provided greater insight into their mechanism of action (57). IMiDs have a direct impact on MM cells by functioning as a molecular glue, binding to a CRL4^CRBN^ E3 ubiquitin ligase (cereblon) and leading to degradation of neo-substrates including ikaros (IKZF1) and aiolos (IKZF3). This results in downregulation of IRF4/MYC and MM cell death (58, 59).

## 6 Immunotherapies as a second revolution in multiple myeloma treatment

**Figure 2:**
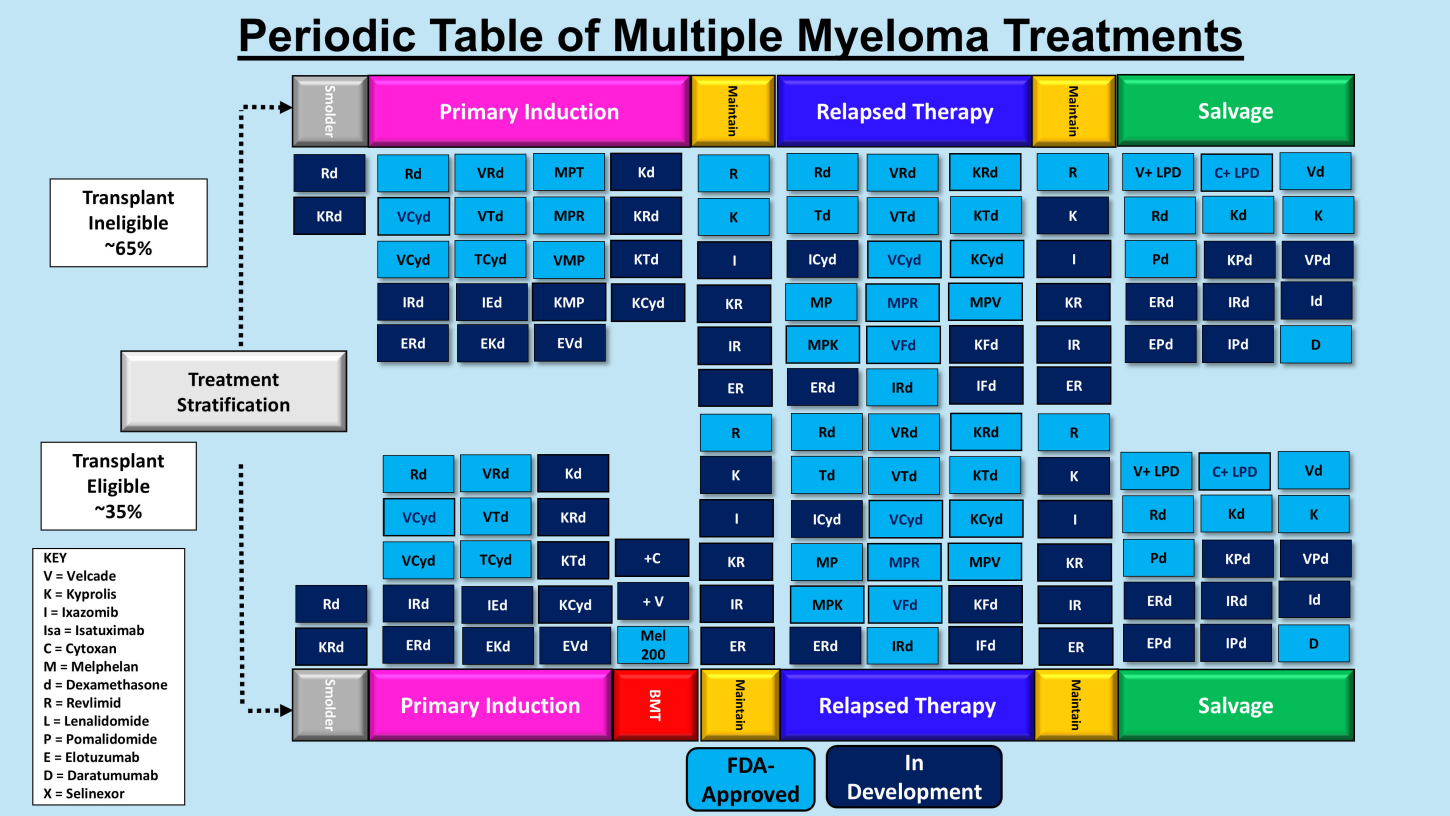
Periodic table of multiple myeloma treatments. Shown is a table of the agents used for transplant eligible and ineligible patients in the pre-immunotherapy era.

**Figure 3:**
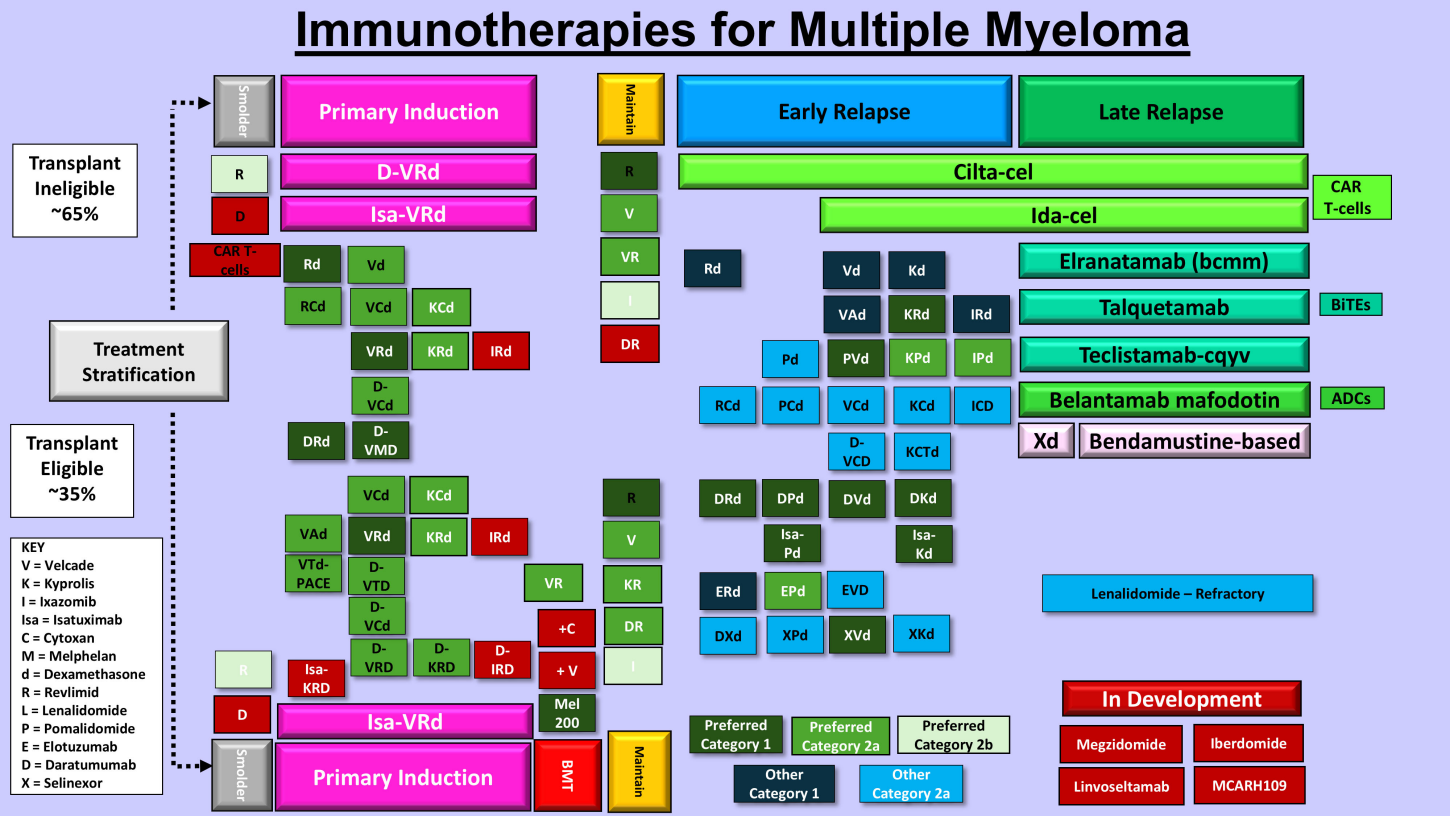
Immunotherapies for multiple myeloma treatment. Shown are the current FDA-approved and investigational immunotherapy agents for ND and RRMM.

A number of recent strategies have been developed to revitalize a patient’s innate immune response towards cancer cells (**Figures 2**, **3**). One of the earliest pieces of evidence of the role of immunotherapy in MM was the introduction of allogeneic (allo)-hematopoietic stem cell transplantation (HCT). HCT offers potential for long-term remission and possible curative outcome in a patient subset through potent graft-versus-myeloma (GVM) effects. Anti-tumor activity is generated through donor-derived alloreactive lymphocytes that target cancer cells, and additional benefit from donor lymphocyte infusions for post-transplant relapse (60). Allo-HCT is limited by harmful toxicities, e.g., graft-versus-host disease (GVHD), infections, combined with significant treatment-related mortality (TRM) (61). To broaden patient eligibility, reduced-intensity conditioning (RIC) regimens were evaluated which led to lower early morbidity countered by increased rates of disease relapse. GVM activity highlights the immune system’s remarkable ability to cure a subset of patients, pushing the role of immunotherapy. Additional modalities have subsequently been introduced to harness the host immune system, e.g., targeted monoclonal antibodies (mAbs), chimeric antigen receptor (CAR) targeting T-cells, and bispecific T-cell engagers (BiTEs).

### 6.1 Monoclonal antibodies in ND and RRMM

The US FDA has authorized three mAbs for the treatment of MM patients. Daratumumab (Dara) and isatuximab (Isa) both target CD38, while elotuzumab (Elo) targets the surface glycoprotein signaling lymphocytic activation molecule F7, SLAMF7/CS1, in the CD2 Ig superfamily (IgSF) subset (62–67). Elo directly activates NK cells and mediates ADCC through the CD16 pathway to yield antimyeloma activity. Anti-CD38 mAbs function through various mechanisms that include complement-dependent cytotoxicity (CDC), antibody-dependent cell-mediated cytotoxicity (ADCC), and antibody-dependent cell-mediated phagocytosis (ADCP). CD38 mAbs can also induce programmed cell death, reduce mitochondrial transfer, inhibit adenosine production and adhesion molecule function, and regulate enzyme activity to induce the death of MM cells.

Dara was FDA-approved in 2016 as a breakthrough orphan drug based on two independent randomized trials in the relapsed setting. The FDA approved Dara-hyaluronidase-fihj (Darzalex Faspro) in combination with bortezomib, Len and Dex for induction and consolidation in patients with ASCT-eligible NDMM patients, based on the Perseus study (NCT03710603), which became the standard induction regimen for ND patients (68). The addition of Dara to a triplet induction regimen of VRd (bortezomib, Len and Dex) significantly improved PFS, and yielded an 84% 48-month PFS with minimal residual disease (MRD) negativity in transplant-eligible individuals.

The FDA has approved Isa-irfc**<b> </b>**(Sarclisa, Sanofi-Aventis) in combinations for patients with RRMM. Isatuximab-irfc**<b> </b>**has been authorized in combination with bortezomib, Len, and Dex (VRd) for NDMM, ASCT-ineligible individuals based on the IMROZ trial, which became the standard induction regimen for this patient population (69). Isatuximab added to VRd for transplant-ineligible patients enhanced MRD negativity and yielded a 48-month PFS rate ~70%. ICARIA-MM was a randomized, multicenter, open-label, phase 3 study of patients (aged ≥18 years) with RRMM who had received >2 previous lines of therapy (LOT), including Len and a PI, and had an ECOG PS of 0–2. Isa+POM/Dex resulted in a 6·9-month difference in median OS compared with POM/Dex and is a new standard of care for Len-refractory and PI-refractory/relapsed MM (65).

Elo has been approved by the FDA for use in combination with Len and Dex in patients with RRMM. Elo is effective as a single agent, as well as in combinations, supporting the use of Elo in NDMM patients (70, 71). ELOQUENT-1 was an open-label, multicenter, randomized, phase 3 trial conducted at 185 sites in 19 countries. Eligible patients were aged >18 years with ND, untreated, symptomatic myeloma and not candidates for high-dose therapy plus HSCT, and an Eastern Cooperative Oncology Group (ECOG) performance status of <2. Patients were randomly assigned (1:1) to receive Elo+Len/Dex or Len/Dex using an interactive voice response system, stratified by the International Staging System (ISS; stage I-II *vs* III), age (<75 years *vs* ≥75 years), and ECOG performance status (0 *vs* 1-2). In both treatment groups, patients received 25 mg Len orally on days 1–21 of each cycle and 40 mg Dex on days 1, 8, 15, and 22 of each cycle. The primary endpoint was PFS. Median PFS was not significantly different between the groups: 31·4 months (95% CI 26·2-36·8) in the Elo+Len/Dex group versus 29·5 months (23·5-34·3) in the Len/Dex group (HR 0·93, 95·71% CI 0·77-1·12; stratified log-rank p=0·44). The median follow-up was 70·6 months (IQR 35·1-79·2). The most common grade 3–4 treatment-related adverse event was neutropenia (64 [17%] of 371 *vs* 79 [21%] of 371). Study drug toxicity was the reported cause of death in five (1%) *vs*. four (1%) patients. Elo+Len/Dex did not significantly improve PFS *vs*. Len/Dex in patients with NDMM who are ineligible for HSCT.

In the phase III ELOQUENT 2 trial, the addition of Elo to Len/Dex (ERd) led to superior ORR, PFS and OS compared to Len/Dex in patients who had received 1–3 prior LOT. In the randomized phase II ELOQUENT 3 trial, the addition of Elo to Pom/Dex (EPd) led to superior ORR, PFS and OS compared to Pom/Dex in patients with RRMM, ~60% of which had only received ≤3 prior LOT. In ELOQUENT 2, patients were not previously treated with Dara and in ELOQUENT 3, <5% of patients were previously treated with Dara. Retrospective data suggest that the efficacy of Elo is diminished with prior Dara exposure (72). Current frontline treatment of MM often includes Len and Dara, with treatment administered until disease progression. There is no singular standard of care for patient’s refractory to these drugs at time of relapse. The results of a recent study suggest that EPd is an active and well-tolerated regimen in RRMM, even in real-world patients. Furthermore, EPd may be useful, especially in Dara-naïve patients (73).

### 6.2 CAR T-cells

In 1987, the immunologist Yoshikazu Kurosawa proposed the notion of a chimeric

T-cell receptor (TCR) which combined the antibody derived variable regions (VH/VL) with constant portions derived from the TCR (74). In 1989, Zelig Eshhar described a strategy to redirect T-cells to recognize antigens in a non-major histocompatibility complex (MHC)-restricted manner (75). Eshhar designed synthetic type I receptors that consisted of an extracellular Ag recognition, transmembrane, and an intracellular signaling domain to activate T-cells (76). Following transfection of these constructs, originally termed “T-bodies”, into a cytotoxic T-cell hybridoma, expression of a functional TCR was detected and the chimeric TCR exhibited the idiotope of the Sp6 anti-TNP Ab. The strategy provided T-cells with a non-MHC-restricted reaction to the given hapten. Transfectants specifically produced Il-2 and elicited cytotoxicity in response to TNP-bearing target cells.

Autologous T-cells were genetically modified to express a 2^nd^-generation CAR 19-28z, specific to the B-cell lineage CD19 and demonstrated efficacy in chemotherapy-refractory chronic lymphocytic leukemia (CLL) or relapsed B-cell acute lymphoblastic leukemia (ALL) patients (77). The short-term persistence of infused T-cells was enhanced by antecedent administration of cyclophosphamide and was inversely proportional to tumor burden within the peripheral blood. Patient T-cells were engineered to express not only CD19 but also costimulatory molecule CD28 joined with a CD3ζ chain as a 2^nd^ generation CAR T-cell (78, 79). The 2^nd^-generation CAR T-cells were well tolerated, although there was notable cytokine release that correlated with burden of disease. Treated patients achieved tumor eradication and complete remission (CR).

Idecabtagene vicleucel (ide-cel) and ciltacabtagene autoleucel (cilta-cel) have been authorized by the US FDA for the treatment of MM patients following multiple prior LOT that included an IMiD, PI, and anti-CD38 monoclonal antibody (**Table 1**) (86, 93–101). The European Medicines Agency (EMA) likewise has endorsed these chimeric engineered T-cell therapies for MM patients following >3 prior LOT. While both cellular therapies target BCMA, they differ in their engineered TCR design, efficacy, and side effect profiles (**Table 1**). Ide-cel harbors a single BCMA-binding domain, while cilta-cel employs two distinct BCMA-binding regions to increase its binding affinity that may potentially contribute to higher response rates (86, 93–99). Ide-cel demonstrated efficacy in the KarMMa trial in which heavily pre-treated RRMM patients were enrolled. Ide-cel achieved an ORR of 73%, with 33% of patients achieving a CR and a median PFS of 8.8 months. However, relapses were frequent and underscored the need for more durable immune-based therapies and CAR T-cell designs. Cilta-cel achieved even more promising results in the Cartitude-1 trial yielding an ORR of 98% with 80% of patients deemed a stringent CR (sCR). Deep and durable responses were observed with cilta-cel to suggest it may offer long-term benefit. KarMMa-3 significantly improved PFS to 13.3 months with ide-cel to 4.4 months observed with standard regimens. Cartitude-4 demonstrated that cilta-cel achieved higher efficacy, with a PFS not yet reached and an ORR of 84%, including 71% of patients achieving MRD negativity (100, 101).

**Table 1:** Comparison of the FDA-approved CAR T-cells for the treatment of relapsed and/or refractory multiple myeloma.

	Idecabtagene vicleucel (80, 81)	Ciltacabtagene autoleucel (82, 83)
Target Protein	BCMA (extracellular domain)	BCMA (extracellular domain) (84)
scFv Binding Protein / Targeting Single Domain Antibodies	Murine (85)	Cameloid (Berdeja et al., 2021 (86)m San-Miguel et al., 2023 (87))
Generation Type	Second generation (85)	Second generation (83, 85)
Ratio of CD8:CD4 at time of infusion	0.820138889	0.820833333
Ratio of CD8:CD4 at post-infusion	N/A	0.820833333
T-Cell lifespan – average range	14 - 20 months (81)	12 - 18 months (88)
Peak T-Cell Level- copies/μg cfDNA	>10^5^ (89)	4.8 x 10^4^ **(copies/µg genomic deoxyribonucleic acid (gDNA)** (90)
CAR T-cell dose	150 - 450 x 10**^6^** (91)	0.75 x 16**^6^** / kg Body weight (91)
Vein-to-vein time (range)	47-71 days (92)	47-71 days (92)
Route of administration	One time IV infusion (84)	One time IV infusion (82, 83)

CAR T –cell product	Idecabtagene vicleucel				Ciltacabtagene autoleucel			
Clinical trial(s)	KarMMa-1 (80, 81)	KarMMa-3 (80, 81)	Real World		CARTITUDE-1^118^	CARTITUDE-4^119^	Real World	
Trial phase	II	III			Ib/II	III		
Patient number (n)	128	254	108 [13]	603 [14]	97	208	139 [15]	18 Center [16]
Median age (years)	61	63	64	65	61	62	64	67.7
LOT n (range)	6 (3–16)	3 (2–4)	N/A	N/A	6 (3–18)	2 (1–3)	N/A	
Triple class exposed	N/A	N/A	N/A	N/A	N/A	53 (25.5)	N/A	17 (94.4)
Triple class refractory-no.(%)	108 (84)	164 (65)	N/A	N/A	85 (88)	30 (14.4)	N/A	N/A
Penta drug exposed-no.(%)	77 (60)	N/A	N/A	N/A	81 (84)	14 (6.7)	N/A	N/A
Penta drug refractory- no.(%)	33 (26)	15 (6)	-41	-36	41 (42)	2 (1.0)	-36	N/A
High risk cytogenetics- no.(%)	45 (35)	107 (42)	-33	(45) ‡	23 (24)	-59.4	-41	N/A
Extramedullary disease- no.(%)	50 (39)	61 (24)	-53	-17	13 (13)	N/A	-35	N/A
Plasma cell leukemia-no.(%)	N/A	1 (<1) *	N/A	-1.5	N/A	N/A	N/A	N/A
Lymphoid depletion regimen	Fludarabine (30 mg per square meter of BSA per day) and cyclophosphamide (300 mg per square meter per day) for 3 consecutive days followed by 2 days of rest before ide-cel administration	Fludarabine (30 mg per square meter BSA area per day) and cyclophosphamide (300 mg per square meter per day) for 3 consecutive days, followed by 2 days of no treatment before administration	N/A	Fludarabine in combination with cyclophosphamide in 95% (n=574) of patients (dosage not provided).	300 mg/m2 of cyclophosphamide and 30 mg/m2 of fludarabine (administered intravenously daily for 3 days) administered 5–7 days after the start of lymphodepletion.	300 mg of cyclophosphamide per square meter of body-surface area and 30 mg of fludarabine per square meter daily for 3 days, administered 5-7åå days before the cilta-cel infusion	Fludarabine and cyclophosphamide: 81%, bendamustine: 11%, Cy: 4%, and cladribine + Cy: 4%”	N/A
Indication	Indicated for the treatment of adult RRMM patients after >2 prior lines of therapy, including an IMiD, proteasome		N/A	Patients had received 6-7 prior lines		Patients had received >1 prior line of therapy, including a proteasome inhibitor and an IMiD, and are refractory to lenalidomide (Revlimid).	N/A	N/A
	inhibitor, and anti-CD38 mAb			of treatment				
Clinical trial(s)	KarMMa-1	KarMMa-3	RWD		CARTITUDE-1	CARTITUDE-4	RWD	
ORR- no.%	94 (73)	181(71)	83†	71	94 (97)	176 (84.6)	-80	17 ( 94.4)
CR or better	42 (33)	98(39)	34†	27	65 (67)	152 (73.1)	-40	
MRD negativity 10^-5^/10^-6^	33 (26)/20 (16)	51 (20)/32 (13)	N/A	N/A	53 (55) / N/A	126 (71.6)****/ N/A	N/A	N/A
DOR months	10.7	14.8	N/A	N/A	N/A	NR 12-month DOR: 85%	N/A	N/A
Time to first response months (range)	1 (0.5–8.8)	2.9 (0.5–13)	N/A	N/A	1 (0.9–10.7)	2.1 (0.9–11.1)	N/A	N/A
PFS (months)	8.8	13.3	N/A	6	NR 27-month PFS: 55%	NR 12-month PFS: 76%	N/A	N/A
OS (months)	19.4	N/A	N/A	6	NR 27-month OS: 70%	NR 12-month OS: 84%	N/A	N/A
Follow-up (months)	13.3	18.6	N/A	N/A	27.7	15.9	N/A	N/A

Product	Idecabtagene vicleucel				Ciltacabtagene autoleucel			
Clinical trial	KarMMa-1	KarMMa-3	RWD		CARTITUDE-1	CARTITUDE-4	RWD
Infections (all grades) (%)	69	58	N/A	N/A	58	62	N/A	N/A
Infections (grade ≥ 3) (%)	22	24	N/A	N/A	20	27	N/A	N/A
CRS (all grades) (%)	84	88	N/A	81	95	76	N/A	12 (67.7)
CRS (grade ≥3) (%)	5	5	N/A	3	4	1	7	0
Median onset of CRS – days (range)	1 (1-12)	1.0 (1.0–14.0)	N/A	2	7(5-8)	8.0 (1–23)	N/A	N/A
Median duration of CRS	5 (1-63)	3.5 (1.0–51.0)	N/A	N/A	4(3-6)***	3.0 (1–17)	N/A	N/A
ICANS (all grades) (%)	N/A	N/A	N/A	N/A	17	5	N/A	2 (11.1)
ICANS (grade ≥3 ) (%)	N/A	N/A	N/A		2	0	8	1 (5.56)
Neurotoxicity (all grades) (%)	18	15	N/A	N/A	21	21	N/A	N/A
Neurotoxicity (grade ≥ 3) (%)	3	3	N/A	N/A	9	3	N/A	N/A
Neuromuscular disorders	N/A	N/A	N/A	N/A	N/A	N/A	N/A	N/A
Neutropenia (grade ≥ 3) (%)	89	76	N/A	N/A	95	90	N/A	N/A
Thrombocytopenia (grade ≥ 3) (%)	52	42	N/A	N/A	60	41	N/A	N/A
Anemia (grade ≥ 3) (%)	60	51	N/A	N/A	68	36	N/A	N/A
Tocilizumab use (%)	52	72	N/A	N/A	69	40	N/A	N/A
Second primary malignancies no.(%)	128 (100)	13 (6)**	N/A	N/A	20	4.3	N/A	N/A
Second primary hematologic malignancies	N/A	3 (1)**	N/A	N/A	10	1.4	N/A	N/A

*From n = 250, those with grade 5 adverse effects.

**Second primary malignancy of safety population (n=125); safety population defined as patients who received ide-cel who were used “in the assessment of treatment-related adverse events, investigator-identified neurotoxic events, and cytokine release syndrome”

***One patient had CRS for 97 days; excluding this outlier, the average range for CRS was 14 days.

****Out of those who had an ORR (n=176).

†RWD on day 30 (as compared to KarMMa-1 and KarMMa-3, whose rates are based on best response).

‡Including Abnormalities of 1q(ab1q).

A common side effect of CAR T-cell therapy is cytokine release syndrome (CRS), which can affect up to 90% of patients. A second complication of CAR T‐cell therapy is immune effector cell‐associated neurotoxicity syndrome (ICANS). Despite superior efficacy, cilta-cel carries a higher risk of neurotoxicity (102–105). ICANS commonly involves cognitive impairment that ranges from acute confusional states to severe encephalopathy. Although immune effector cell‐associated encephalopathy (ICE) scoring is a valid method to assess neurotoxicity, it does not generally capture subtle or early changes in status. In Cartitude-1, 95% of patients experienced CRS with 5% having grade >3 effects. Moreover, a concerning aspect of cilta-cel’s toxicity profile is the incidence of delayed neurotoxicity, e.g., parkinsonism and other movement disorders, which has been observed in up to 12% of patients. Neurotoxic effects can manifest weeks to months post-infusion and have prompted increased vigilance as well as specific mitigation strategies. Conversely, in the KarMMa study, patients exhibited a more manageable safety profile with CRS reported in 84% of patients following the infusion of ide-cel. Neurotoxicity was reported in 18% of patients, with severe cases occurring in ~3%. Acute anaphylaxis events and tumor lysis syndrome (TLS) also occur after CAR T-cell infusion. The relatively manageable neurotoxicity profile of ide-cel makes it an appealing option for the elderly and frail patient populations.

Real-world data have generally supported the results generated the pivotal trial for CAR T- cell therapies to treat MM, with both agents demonstrating high efficacy outside of the controlled setting (106). However, it is increasingly appreciated that patients that are enrolled on clinical trials with strict inclusion and exclusion criteria generally do not represent the average patient seen and treated in the non-academic or outpatient clinical practice. Real-world experiences highlight the challenges of managing the toxicities associated with CAR T-cells, particularly the neurotoxicity observed following cilta-cel. CAR T-cell dysfunction, tumor-intrinsic resistance, and an immunosuppressive tumor microenvironment (TME) remain obstacles that limit CAR T-cell efficacy (101–105). Also, CAR T-cell exhaustion caused by persistent Ag stimulation or tonic CAR signaling leads to gradual loss of function and reduced cytotoxic activity (106–108). Immunosuppressive cells and factors within the TME further also contribute to CAR T-cell exhaustion, exclusion from the tumor bed and loss of functionality.

### 6.3 Bispecific T-cell engagers

BiTes are a specific type of bispecific Ab designed to bridge T-cells and tumor cells, and, consequently, facilitate T-cell-mediated elimination of cancer cells (109–112). BiTEs have two distinct binding domains that can bind simultaneously to either two antigens or two epitopes (antigenic regions) of the same antigen. BiTEs typically bind to a tumor epitope as well as CD3 that is presented on autologous T-cells, leading to T-cell activation and tumor lysis. BiTEs can be considered as next generation mAbs that target antigenic epitopes (of the same or different proteins presented on the same or different cell types, e.g., tumor and T-cells), to elicit multiple downstream physiological or anti-tumor responses. Teclistamab and elranatamab (Elra) are each BiTEs that target BCMA on MM cells and also bind to CD3 presented on T-cells. Talquetamab targets G protein–coupled receptor, family C, group 5, member D (GPRC5D) expressed on MM cells and CD3 present on T-cells. Talquetamab may represent a viable target for patients resistant to BCMA-directed therapy. BiTes and Bispecifics are valuable not just as an alternative to ASCT but also have a role in transplant ineligible patients too.

Teclistamab is referred to as both a BiTE (specific T-cell Engager) and a BsAb (**Bi** specific **Ab**) because it functions as both (109). Teclistamab is a specific type of bispecific antibody that works by binding to two different targets: CD3 on T cells and BCMA on myeloma cells. in the phase 1 dose-defining portion of the study, teclistamab showed promising efficacy in patients with RRMM (109). Teclistamab is the first approved off-the-shelf BCMA×CD3 bispecific antibody for the treatment of patients (pts) with RRMM based on data from the pivotal phase 1/2 MajesTEC-1 study (NCT03145181/NCT04557098). MajesTEC-1 evaluated patients with RRMM after = 3 therapy LOT, including triple-class exposure to an IMiD, a PI, and anti-CD38 Ab, treated with teclistamab. Patients received weekly subcutaneous injection of teclistamab (1.5 mg/kg BW) after step-up doses of 0.06 mg and 0.3 mg/kg. An ORR of 63% was observed with 39.4% of patients achieving CR or better (109). Median PFS was 11.3 months and median DOR was 18.4 months. The FDA granted accelerated approval to teclistamab-cqyv (TECVAYLI; Janssen Biotech) for the treatment of adult patients with RRMM who have received >4 prior LOT. After ~2 y mFU, patients receiving teclistamab demonstrated deep and durable responses regardless of refractory status, with mPFS of 12.5 months and mDOR of 24 months (not reached in those achieving ≥CR). These long-term follow-up data support teclistamab as a safe and effective off-the-shelf BCMA bispecific therapy for pts with RRMM (110).

Elranatamab is a humanized BCMA-CD3 agent administered subcutaneously once weekly. MagnetisMM-3 (phase 2, NCT04649359) demonstrated that RRMM patients who had received >3 prior LOT and received Elra experienced an ORR of 61%, including 35% that achieved CR or better (111). The FDA also granted accelerated approval to elranatamab-bcmm (Elrexfio) for the treatment of patients with RRMM who have previously received >4 LOT, including a PI, an IMiD, and an anti-CD38 monoclonal antibody. The regulatory decision was based upon data which showed that Elra elicited an ORR of 57.7% (95% CI, 47.3%-67.7%) in 97 patients who were naïve to BCMA-directed therapy (110). The CR rate was 25.8%, VGPR rate of 25.8, and a PR rate of 6.2%. Notably, 82% of responders were estimated to continue to respond to treatment for >9 months.

Talquetamab targets the G protein–coupled receptor family C group 5 member D(GPRC5D). The FDA has also granted accelerated approval to talquetamab-tgvs (Talvey, Janssen Biotech, Inc.) for adults with RRMM who have received at least >4 LOT, including a PI, an IMiD, and an anti-CD38 monoclonal antibody (112–114). Efficacy was evaluated in MMY1001 (MonumenTAL-1) (NCT03399799, NCT4634552), a single-arm, open-label, multicenter study that included 187 patients who had previously received at least four prior systemic therapies. Patients received talquetamab-tgvs 0.4 mg/kg subcutaneously weekly, following two step-up doses in the first week of therapy, or talquetamab-tgvs 0.8 mg/kg subcutaneously biweekly (q 2 weeks), following three step-up doses, until disease progression or unacceptable toxicity. The main efficacy outcome measures were ORR and DOR as assessed IMWG criteria.

In MonumenTAL-1, talquetamab exhibited an impressive ORR of 70% in heavily pre-treated RRMM patients with a median PFS of 7.5 months. Based upon the distribution of the target antigen, a unique pattern of GPRC5D-associated AEs have been observed as well as T-cell redirection–associated AEs (115–117). GPRC5D-associated AEs included dermatologic, i.e., rash, non-rash, and nail toxicities, and oral AEs, i.e., dysgeusia, dysphagia, and dry mouth. Talquetamab, while effective, presents unique side effects related to its GPRC5D target, e.g., skin rashes and nail disorders. Infections are a critical issue, with serious cases reported in 30-40% of patients, necessitating rigorous monitoring and supportive care. The AEs underscore the need for careful patient selection. The incidence of CRS and ICANS were consistent with other T-cell redirection therapies. The incidence of high-grade infections was lower than that observed with BCMA-targeting Bispecific Abs, with less frequent use of intravenous immunoglobulin required. GPRC5D-associated AEs were mostly low grade and led to few discontinuations. Increased talquetamab exposure has led to a higher incidence of dysgeusia. Nutritional monitoring and appropriate supplementation were implemented and high caloric shakes were advised to ensure adequate nutritional intake and prevent weight loss. Oral toxicities and weight loss have beenreported as AEs in the MonumenTAL-1, MonumenTAL-2, TRIMM-2, and RedirecTT-1 studies.

The optimal benefit of BiTEs is hampered by an immunosuppressive TME, a hallmark of MM, which limits efficacy and by undesirable adverse events, especially CRS and severe infections (**Table 2**) (52, 80, 81, 118–122).BiTEs are also associated with significant adverse effects. CRS is a common toxicity, occurring in 60-75% of patients across all three BiTEs, though most cases are low grade. Neurotoxicity, generally mild to moderate, also remains a concern, particularly with elranatamab and teclistamab.

**Table 2:** Comparison of the FDA-approved BiTEs for the Treatment of Relapsed and/or Refractory Multiple Myeloma.

	Teclistamab-cqyv			Elranatamab-bcmm			Talquetamab-tgvs		
T Cell Target	CD3			CD3			CD3		
Multiple Myeloma Target	BCMA			BCMA			BCMA https://www.onclive.com/view/fda-approves-talquetamab-for-relapsed-refractory-multiple-myeloma		
Dosing regimen	Day	Dose		Day	Dose		Day	Dose	
Step-up Dosing Schedule	Day 1	Step-up dose 1	0.06 mg/kg	Day 1	Step-up dose 1	12mg	Day 1	Step-up dose 1	0.01 mg/kg
	Day 4	Step-up dose 2	0.3 mg/kg	Day 4	Step-up dose 2	32mg	Day 4	Step-up dose 2	0.06 mg/kg
	Day 7	First treatment dose	1.5 mg/kg	Day 8	First treatment dose	76mg	Day 7	Step-up dose 3/	0.4 mg/kg
								First treatment dose 1	
							Day 10	First treatment dose 2	0.8 mg/kg
Weekly Standard Dosing schedule	Day 14	Subsequent treatment doses	1.5 mg/kg qW	Day 15	Subsequent treatment doses	76mg qW	Day 14	Subsequent treatment doses 1	0.4 mg/kg qW
Biweekly Standard Dosing schedule							Day 24	Subsequent treatment doses 2	0.8 mg/kg q2W
Dose de-escalation	Week 25	Dose de-escalation	1.5 mg/kg q2W	Week 25	Dose de-escalation	76mg q2W			
Route of administration	Subcutaneous			Subcutaneous			Subcutaneously or Intravenously		
Indication	Adult patients with relapsed or refractory multiple myeloma who had previously received at least 3 prior therapies, including a proteasome inhibitor, an immunomodulatory agent, and an anti-CD38 monoclonal antibody, and had not received prior BCMA-targeted therapy.			Adult patients with relapsed or refractory multiple myeloma who have received at least four prior lines of therapy, including a proteasome inhibitor, an immunomodulatory agent, and an anti-CD38 monoclonal antibody.			Adult patients with relapsed or refractory multiple myeloma who have received at least 4 prior lines of therapy, including a proteasome inhibitor, an immunomodulatory agent, and an anti-CD38 antibody.		

Product	Teclistamab-cqyv			Elranatamab-bcmm			Talquetamab-tgvs		
Clinical trial(s)	MajesTEC-1	RWD		MagnetisMM-3		RWD	MonumenTAL-1		RWD
Trial phase	I/II			II		Not Yet Available	I/II		Not Yet Available
n	165	102	123	123			130**/102***		
Median age (years)	64	66.5	67	68			64–65		
LOT n (range)	5 (2–14)	N/A	N/A	5 (2–22)			6 (2–20)		
Triple class exposed	165 (100.0)	N/A	N/A	123 (100)			129 (99)**/101 (99) ***		
Triple class refractory	128 (77.6)	58 (62) Ƒ	113 (92.6)	119 (96.7)			97(75)**/87(85) ***		
Penta drug exposed	116 (70.3)			87 (70.7)			100 (77)**/79(77)***		
Penta drug refractory	50 (30.3)	45(66)»	74 (60.2)	52 (42.3)			33(25)**/36(35)***		
High risk cytogenetics	38/148 (25.7)	34 (62)	39 (36.8)	31 (25.2)			18 (16) ⊥ /14 (16) ♢		
Extramedullary disease	28 (17.0)	20(45)	43 (36.1) ¦	39 (31.7)			42 (32.3)** /15 (14.7)***		
Plasma cell leukemia	N/A	N/A	N/A	N/A			N/A		

Product	Teclistamab-cqyv			Elranatamab-bcmm			Talquetamab-tgvs		
Clinical trial(s)	MajesTEC-1	RWD		MagnetisMM-3		RWD	MonumenTAL-1		RWD
ORR	104 (63.0)	65 (64)	59.3	75 (61)		Not Yet Available	(68) § / (72) ₩		
CR or better	65 (39.4)	29 (28)	N/A	43 (35)			21 § /28 ₩		
MRD negativity 10^-5^/10^-6^	44 (26.7)/ N/A		N/A	(89.7)* / N/A			11¶		
DOR months	18.4	N/A	N/A	NR 12-month DOR: 75%			7.8–10.2		
Time to first response months (range)	1.2 (0.2–5.5)	N/A		1.2 (0.9–7.4)			0.9 (0.2–3.8)–1.2 (0.3–6.8)		
PFS (months)	11.3	N/A	8.7	NR 12-month PFS: 57%			7.5–11.9 months		
OS (months)	18.3	N/A	N/A	NR 15-month OS: 57%			N/A		
Follow-up (months)	14.1	N/A	N/A	14.7			4.2–11.7		

Product	Teclistamab-cqyv			Elranatamab-bcmm			Talquetamab-tgvs		
Clinical trial	MajesTEC-1	RWD		MagnetisMM-3		RWD	MonumenTAL-1		RWD
Infections (all grades) (%)	76	N/A	67 (54.5)	70			34-47		Not Yet Available
Infections (grade ≥ 3) (%)	45	N/A	33 (26.8)	40			7		
CRS (all grades) (%)	72	N/A	72 (58.5)	58			77–80		
CRS (grade ≥3) (%)	1	N/A	2 (1.6)	0			3–5		
Median onset of CRS - days (range)	2 (1–6)	N/A		2 (1–9)**			2		
Median duration of CRS (days)	2 (1–9)	N/A		2 (1–19)**			2 (1-7)		
ICANS (all grades) (%)	3	N/A	9(7.3)	3			N/A		
ICANS (grade ≥3 ) (%)	0	N/A	1 (0.8)	0			N/A		
Neurotoxicity (all grades) (%)	15	N/A		17			6–8		
Neurotoxicity (grade ≥ 3) (%)	1	N/A		1			0–3		
Neuromuscular disorders	N/A	N/A		N/A			N/A		
Neutropenia (grade ≥ 3) (%)	64	N/A	46 (38.0)	49			26–60		
Thrombocytopenia (grade ≥ 3) (%)	21	N/A	32(26.0)	24			11–23		
Anemia (grade ≥ 3) (%)	37	N/A	39 (31.7)	37			23–33		
Tocilizumab use (%)	36	N/A		23			54–65		
Second primary malignancies	N/A	N/A		N/A			N/A		
Second primary hematologic	N/A	N/A	N/A	N/A			N/A		

* MRD negativity is out in patients with ≥CR and who were evaluable for MRD (n = 29)

** Administration was done subcutaneous and intravenously in this trial, with n=130 being those who received Talquetamab subcutaneously

*** Administration was done subcutaneous and intravenously in this trial, with n=102 being those who received Talquetamab intravenously

⊥ n total = 112

♢ n total = 18

§ Among the patients who received the most active subcutaneous doses (135, 405, and 800 μg per kilogram weekly and 800 and 1200 μg per kilogram every other week)

₩ Among those who received the most active intravenous doses (20 to 180 μg per kilogram weekly)

¶ Among 16 patients with samples available for analysis of minimal residual disease

RWD:

Ƒ Out of n=94; » Out of n=68; ø Out of n=55; ® Out of n=44; ¤ Out of n= 106; ¦ Out of n=119

* Out of patients who were treated with Teclistamab-cqyv and had available cytogenetic data (n=148).

** Relative to the most recent dose as 98.8% of CRS events occurred with the first three doses and 90.6% occurred with the step-up dose.

ASCT has been the standard of care patients with MM for the past three decades. However, high-risk patients still have poor outcomes. Many bispecific therapies are currently under investigation for the treatment of myeloma, e.g., blinatumomab, teclistamab, talquetamab and cevostamab, as well as CAR T-cell therapy using ide-cel and cilta-cel. With CAR-T and bispecific antibodies demonstrating deep and sustained remissions, the role of ASCT in the future treatment of myeloma has become a subject of debate, especially in the frail and elderly populations.

### 6.4 Antibody drug conjugates

ADCs are composed of a mAb that has been covalently linked to a cytotoxic chemotherapy drug, e.g., calicheamicin, monomethyl auristatin E, and microtubule inhibitor MMAF. The ADC is internalized by the tumor cell and then releases the cytotoxic drug, with the cancer cell to minimize damage to neighboring healthy cells. ADCs combine advantages of highly specific targeting ability and highly potent killing effect to achieve the accurate and efficient elimination of cancer cells. ADCs also improve the delivery of cytotoxic drugs to cancer cells and certain ADCs have exhibited improved efficacy over conventional chemotherapy. Belantamab mafodotin consists of a humanized IgG mAb linked to the microtubule-disrupting cytotoxic agent monomethyl auristatin, and targets BCMA (82–84). Initially approved for the treatment of RRMM, belantamab mafodotin was withdrawn from US and European markets following disappointing trial results.

DREAMM-1 and DREAMM-2 demonstrated promising activity of belantamab mafodotin in heavily pretreated RRMM populations (83, 85). DREAMM-1 reported an ORR of 60%, while DREAMM-2 exhibited an ORR of 30% at the approved dose of 2.5 mg/kg. The phase 3 DREAMM-3 study compared belantamab mafodotin to the combination of POM and Dex (Pd) and failed to demonstrate PFS or OS benefit. The phase 3 DREAMM-7 head-to-head trial evaluated the efficacy and safety of BVd triplet *vs* the standard of care triplet DVd, in patients with RRMM treated with ≥1 prior LOT. Belantamab mafodotin was dosed at 2.5mg/kg intravenously q3 weeks and BVd significantly improved PFS compared to DVd (37 *vs*. 13 months) but was associated with higher rates of serious AEs and drug discontinuation due to toxicity. DREAMM-8 investigated belantamab mafodotin with Pom and Dex (BPd) *vs*. bortezomib, Pom, and Dex (PVd). BPd improved PFS at 12 months (71% *vs*. 51%) but again exhibited higher rates of = grade 3 AEs, particularly ocular toxicity. Ocular toxicity is a significant AE associated with belantamab mafodotin caused by the accumulation of the cytotoxic payload in the cornea, leading to blurred vision, dry eyes, corneal ulceration, and, potentially, loss of vision. Ophthalmic monitoring is required before each dose. While ocular AEs can be managed with dose adjustments or temporary treatment holds, they remain a considerable burden for patients and healthcare providers. Other AEs of belantamab mafodotin include thrombocytopenia, nausea, pyrexia, infusion-related reactions, and fatigue.

## 7 Antimyeloma drugs in development

Cereblon E3 ligase modulatory drugs (CELMoDs) are an emerging new class of medications being studied in clinical trials for MM treatment (88, 89). CELMoDs build on the well-established platform of IMiDs and are designed not only target myeloma cells directly but also by engaging other immune cells. CELMoDs offer promise for those who have relapsed after treatment with IMiDs. Two promising orally-available CELMoDs in clinical trials are iberdomide and mezigdomide. Marizomib (MRZ) is a novel, irreversible proteasome inhibitor in clinical development for the treatment of relapsed or relapsed and refractory multiple myeloma (RRMM) (90). MRZ inhibits the 3 proteolytic activities of the 20S proteasome with specificity distinct from bortezomib and carfilzomib. Histone deacetylase (HDAC) inhibition improves the efficacy of proteasome inhibition for multiple myeloma but adds substantial toxicity. Preclinical models suggest that the observed synergy is due to the role of HDAC6 in mediating resistance to proteasome inhibition via the aggresome/autophagy pathway of protein degradation. A phase I/II trial of the HDAC6-selective inhibitor ricolinostat to define the safety, preliminary efficacy, and recommended phase II dose in combination with standard PI therapy (91). Patients with RRMM received oral ricolinostat on days 1–5 and 8–12 of each 21-day cycle. The ORR in combination with daily ricolinostat at ≥160 mg was 37%. The response rate to combination therapy among bortezomib-refractory patients was 14%. At the recommended phase II dose of ricolinostat of 160 mg daily, the combination with bortezomib and Dex is safe, well-tolerated, and active, suggesting that selective inhibition of HDAC6 is a promising approach to MM therapy.

There remain many challenges that warrant further development of CAR T structure and function as they relate to durability of response, T-cell exhaustion due to tonic signaling, immunogenicity, manufacturing-related limitations, and incidence of serious adverse events including cytokine release syndrome and immune effector cell associated neurotoxicity syndrome. D-domain based CAR T-cells are a new class of structurally and functionally distinct cell therapies that represent an alternative to conventional single-chain variable fragment based chimeric antigen receptor T-cells (92). Preclinical studies of a D-domain based targeting BCMA (CART-ddBCMA) have demonstrated effective anti-tumor response in both *in vitro* and tumor models. These studies currently support a first-in-human clinical study of CART-ddBCMA in MM patients. Durvalumab, a PD-L1 inhibitor, is being explored for its potential in treating MM. It works by blocking the PD-L1 protein, which helps cancer cells evade the immune system, thereby potentially enhancing the body’s ability to fight the disease (123). Durvalumab is often combined with other therapies, such

as like Len and Pom, and also with other targeted therapies like Dara.

## 8 Emerging combinations in myeloma therapy

Triplet regimens that consist of a PI, an IMiD, and Dex have been considered a standard of care for patients with NDMM based on RCTs demonstrating improved DOR and long-term PFS and OS compared with doublets (124). Quadruplet regimens have been investigated for NDMM in clinical trials evaluating a PI, IMiD, Dex, and anti-CD38 mAb. The results have shown improvement in increasing the DOR, including MRD negativity rates as well as PFS in patients with NDMM. The US FDA has approved a powerful four-drug combination therapy for frontline treatment of a regimen of Dara, bortezomib, Len and Dex for MM patients (68, 125–127). Disease progression in the first year post-QUADs is uncommon. Currently, there is a lack of high-level evidence to guide the management of transplant-eligible patients with NDMM who experience disease relapse after up-front quadruplet induction. Ravi et al. analyzed a large and mature institutional dataset of patients with NDMM treated with QUADs with the intent to proceed with ASCT (128). Similarly, a recent Mayo Clinic study reported that 6.7% of patients with NDMM had treatment refractoriness to initial induction therapy (129). Patients who did not respond to initial induction therapy had a poor median PFS of 4.2 months, compared to 50.8 months in patients who were able to achieve a complete response to initial induction therapy. Understanding the mechanisms of resistance to anti-myeloma therapy is an ongoing challenge. Efforts should focus on the early deployment of therapies with new mechanism of action for patients experiencing treatment failure after QUADs.

The FDA approved an injectable form of the drug Dara that includes hyaluronidase (Darzalex Faspro) given in combination with bortezomib, Len, and Dex for patients who are eligible for ASCT. The FDA has also approved isatuximab (Sarclisa) given with the same three drugs for patients newly diagnosed with MM who are ineligible for SCT. Because of the lengthy process required to treat a patient with CAR T-cells, bridging therapy (BT), administered after leukapheresis but prior to CAR T-cell infusion, has become an important component of safely administering CAR T therapy. Establishing a strategy for sequencing of T cell-redirecting therapies for RRMM also represents a pressing clinical need. The clinical and immunologic impact of bispecific T cell-engaging BsAb as BT to subsequent BCMA-directed CAR T-cell therapies in 52 patients with RRMM was recently evaluated. BsAbs were shown to be a potent and safe option for BT, achieving the highest ORR (100%) to BT compared with chemotherapy, anti-CD38, or anti-SLAMF7 antibody-based regimens (46%) (130).

## 9 Conclusions

The treatment paradigm for MM has evolved from non-specific agents to conventional chemotherapeutics to targeted agents generated based upon the biology of disease and more recently immunotherapy (41, 128, 131–133). Improved understanding of myelomagenesis and the biology of disease has given rise to new actionable targets for therapies that stimulate the immune system to effectuate long-lived tumor destruction (134, 135). Many of these pharmacologics are the pillars of present-day frontline treatment regimens, but are restricted by tight therapeutic indices, undesirable toxicities and significant AEs, combined with frequently acquired drug resistance.

Many challenges limit the therapeutic efficacy of CAR-T cells in hematological malignancies (86, 87, 96, 136–146). Barriers to effective CAR T-cell therapy include severe life-threatening toxicities, modest anti-tumor activity, antigen escape, restricted trafficking, and limited tumor infiltration. In addition, the host and tumor microenvironment interactions with CAR-T cells critically alter CAR-T cell function. Furthermore, a complex workforce is required to develop and implement these treatments. Lengthy waitlists to receive CAR T-cells introduce a pre-apheresis, patient selection bias. Post-apheresis patient dropout introduces a second layer of bias and further complicates the interpretation of treatment efficacy. Similar challenges also complicate the comparison of CAR T-cells with off-the-shelf agents, e.g., BsAbs and allogeneic CAR T-cell products (147–150). At present, both BsAbs and CAR T-cells are approved by the US FDA and are available as standard of care therapy. Certain patients may be better served by BsAbs that afford immediate access and a consequent reduced risk of disease progression or death while waiting for apheresis and CAR T-cell infusion. Patients who encounter manufacturing failure would have an even greater risk of compromised BM reserve and T-cell health and are more susceptible to cytopenia and life-threatening infections. Second, all patients in the pivotal KarMMA trial were required to be refractory to the last line of regimen before CAR T-cell therapy. However, in the study by Hansen et al, ~two-thirds of patients had refractory disease.

Selecting and sequencing immunotherapies in the myeloma setting presents a challenge, especially when lacking substantial historical data and necessitates the consideration of ECOG status, previous and potential AEs and disease resistance mechanisms. When sequencing BCMA-directed CAR T-cells and bispecific agents, clinicians must be aware of the effect that prior therapies may have on the efficacy of subsequent agent. Financial toxicity is another challenge since CAR T-cells and BiTEs are expensive and treatment decisions lead to healthcare disparities and raise concerns regarding long-term sustainability and access (86, 96, 137). Future myeloma care will undoubtedly continue to evolve over the next century to improve OS, access to care and quality of life. Ongoing research strives to identify targets that prevent or overcome drug resistance and advance precision oncology treatment approaches to tailors a patient’s care based on unique tumor and immune cell characteristics, e.g., mutations, genomics, T-cell profile. An approach focused on deciphering individual patient tumor genetics and MHC class I antigenic profiles will influence the parallel development of personalized (CAR) T-cell therapies and cancer vaccines.
